# The Heat Shock Protein HSP70 Promotes Th17 Genes’ Expression via Specific Regulation of microRNA

**DOI:** 10.3390/ijms21082823

**Published:** 2020-04-17

**Authors:** Hanna Cwiklinska, Maria Cichalewska-Studzinska, Krzysztof W. Selmaj, Marcin P. Mycko

**Affiliations:** 1 Department of Neurology, Laboratory of Neuroimmunology, Medical University of Lodz, Pomorska 251, 92-213 Lodz, Poland; h.cwiklinska@gmail.com (H.C.); daszuda@gmail.com (M.C.-S.); 2 Department of Neurology, Laboratory of Neuroimmunology, Faculty of Medicine, University of Warmia and Mazury in Olsztyn, Warszawska 30, 10-082 Olsztyn, Poland; strongagonist@gmail.com

**Keywords:** HSP70, microRNA, Th17, autoimmune demyelination, experimental autoimmune encephalomyelitis, stress response

## Abstract

T helper cells type 17 (Th17) are orchestrators of autoimmune conditions, including multiple sclerosis (MS), but mechanisms of Th17 pathogenicity remain unknown. MicroRNAs (miRNA) are known to control T cells. To understand the function of miRNA in Th17, we have established a T cell line, EL4-TCR^+^, that resembles the expression pattern of the Th17 cells. Subsequently, we have evaluated the crosstalk between miRNA and Th17 genes’ expression using a combination of gene expression profiling, gene expression manipulation, RNA and protein immunoprecipitation, as well as confocal microscopy. We have found that Th17-related miRNA were strongly expressed in EL4-TCR^+^ cells following the binding of the cluster of differentiation 3 (CD3) component of the T cell receptor (TCR). Furthermore, a specific inhibition of these miRNA resulted in downregulation of the critical Th17 genes’ expression. Surprisingly, this mechanism relied on the function of the stress signal regulator heat shock protein 70 (HSP70). Upon activation, HSP70 co-localized intracellularly with miRNA processing proteins. Precipitation of HSP70 resulted in enrichment of the Th17-associated miRNA. Finally, HSP70 inhibition led to downregulation of the Th17 genes’ expression and ameliorated development of autoimmune demyelination. Our study demonstrated that HSP70 facilitates specific miRNA function leading to Th17 genes’ expression, a mechanism linking stress and autoimmunity.

## 1. Introduction

Multiple sclerosis (MS) is an organ-specific autoimmune disease manifested by chronic inflammatory demyelination of the central nervous system (CNS) [1]. T helper type 17 (Th17) cells are characterized by expression of the transcription factors retinoic acid receptor-related orphan receptor alpha and gamma t (ROR-alpha and ROR-yt) and production of proinflammatory cytokines, including the interleukin (IL)-17 family. Th17 play a key role in defense against extracellular pathogens, but are also pivotal for the propagation of autoimmune diseases, especially autoimmune demyelination [2]. Th17-driven autoimmunity against a putative myelin autoantigen, has been proposed as one of the most important aspects of MS pathogenesis, especially for the early initiation of disease [3]. This has been predominantly supported by research on the MS animal model, experimental autoimmune encephalomyelitis (EAE). Mice with impaired numbers or function of Th17 cells, particularly mice deficient in the cytokines IL-6 or IL-23, are largely resistant to EAE [2,3,4]. However, the precise mechanisms that govern the development and function of pathogenic Th17 cells resulting in autoimmune demyelination are still being discovered [5]. Thus, Th17-targeting therapeutic approaches for MS are still far from being established.

MicroRNAs (miRNA) operate as short non-coding RNA molecules that are processed from larger transcripts of non-classical genes by Drosha and Dicer nucleases [6]. Mature miRNA biogenesis is a tightly controlled multistep process, finalizing in the production of an approximately 22 nucleotide long duplex. miRNA regulate gene-expression programs by reducing the translation and stability of target mRNAs [7]. The immature miRNA duplex is transferred to one of the Argonaute (AGO) proteins and with a leading strand of miRNA guiding the AGO to interact with the target mRNA. These key players of the miRNA pathway, like Drosha, Dicer and AGO, are complemented by a number of the proteins forming a RNA-induced silencing complex (RISC), an essential structure conducting miRNA function. It has been estimated that expression of as many as one third of protein-coding genes may be regulated by miRNA [8], and the details of this effect still need to be fully elucidated. This includes an investigation into the systems that regulate activity of miRNA, including both proteins and non-coding RNA transcripts [9].

Many miRNAs have emerged as important factors in Th subtypes development and differentiation. To this end, we and others have demonstrated that miRNA, like miR-301a, miR-326, miR-146a and miR-155, act as critical regulators of the Th17 program [10,11,12,13,14,15]. These studies have also highlighted a number of the molecules that mediate the impact of miRNA on Th17’s fate, revealing a complexity of different pathways leading to the development of this Th subtype. Furthermore, manipulation of these miRNA’s function has been found to affect the in vivo development of autoimmune demyelination. With this promise, a further investigation into a general paradigm of how miRNAs operate in promoting a Th17 population that results in development of autoimmune demyelination is likely to provide a new potential targets for a therapeutic intervention.

Heat shock protein 70 (HSP70) is a highly evolutionarily conserved cytoplasmic and nuclear protein that functions in various intracellular processes and as a molecular chaperone for damaged intracellular proteins [16]. Its role has been particularly implicated in the host response to a wide variety of stressors, including infection, injury, oxidative damage, hypoxia and thermal stress. HSP70 has been shown to promote protein folding/refolding, prevent aggregation of proteins by targeting misfolded proteins for degradation by the proteasome and to facilitate protein transport [17,18]. With these properties, HSP70 is believed to contribute significantly to a cellular protection mechanism called the "protein triage" [19]. We have already demonstrated a significance of the HSP70 during MS, especially a presence in Th cells for the development of the Th17 responses and promotion of autoimmune demyelination [20,21,22]. To further study the mechanism of HSP70 involvement in the Th17 program, we have utilized a murine cell line with a natural Th17-type profile. With this model, we have confirmed that HSP70 is directly linked to the Th17 transcriptional program and helps to stabilize it. Interestingly, HSP70 has been revealed as an instrumental factor in the process of the generation of Th17-promoting miRNA.

## 2. Results

### 2.1. EL4 TCR-Positive Cells Stimulation Induces Expression of Th17 Marker Genes

**Figure 1:**
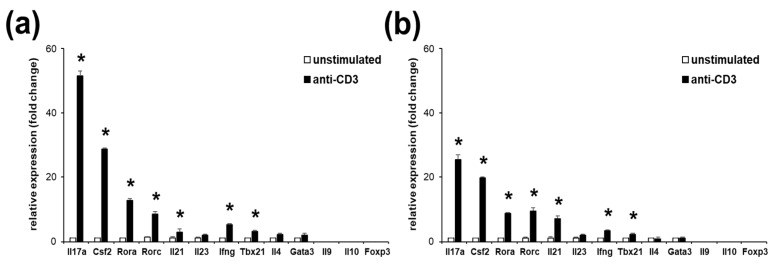
EL4 TCR^+^ cells upregulate T helper cells (Th) type 17 genes in a manner similar to murine Th17 cells. Gene expression analysis of EL4 TCR^+^ cells **(a)** or Th17 polarized murine cells following an anti-CD3 stimulation (**b**). Il17a, Csf2, Rora, Rorc, Il21 and Il23 are markers specific for Th17. Ifng and Tbx 21 are markers of Th1, Il4 and Gata3 are markers of Th2, Il9, Foxp3 and Il10 are markers of Th9, Treg and Tr1, respectively. Data represent mean +/− standard deviation (SD). * *p* < 0.05, Student’s *t*-test.

It is known that mouse lymphoma EL4 cells, when activated, overexpress the IL-17 family of cytokines [23,24,25]. We have found that a minority of EL4 cells are expressing TCR and CD3 (not shown). Therefore, to enhance the gene expression response of the EL4 cells to the stimulation, we sorted and subcloned TCR-positive cells (EL4 TCR^+^). Subsequently, we have stimulated these cells and assayed the gene expression pattern with regard to the presence of the Th marker genes. We have found that TCR activation via binding of the CD3 component using anti-CD3 antibodies of EL4 TCR^+^ cells resulted in a significant, strong induction of expression of Il17a, Csf2, Rora, Rorc and to lesser degree, Il21 (all Th17 markers), and two Th1 marker genes, Ifng and Tbx21 (Figure 1a). Th2, Th9, Treg or Type 1 regulatory (Tr1) T cells signature genes and Il23 were not significantly induced in EL4 TCR^+^ cells after TCR stimulation. Interestingly, the pattern and range of the Th-specific genes’ upregulation in EL4 TCR^+^ cells resembled gene expression changes observed in the murine Th17 cells following their TCR stimulation (Figure 1b). Thus, we have found that EL4 TCR^+^ cells’ responses mimic a Th17 cell in their reaction to TCR activation via binding of CD3.

### 2.2. HSP70 Inhibition Reduces Marker Gene Expressions of Th17 Cells

**Figure 2:**
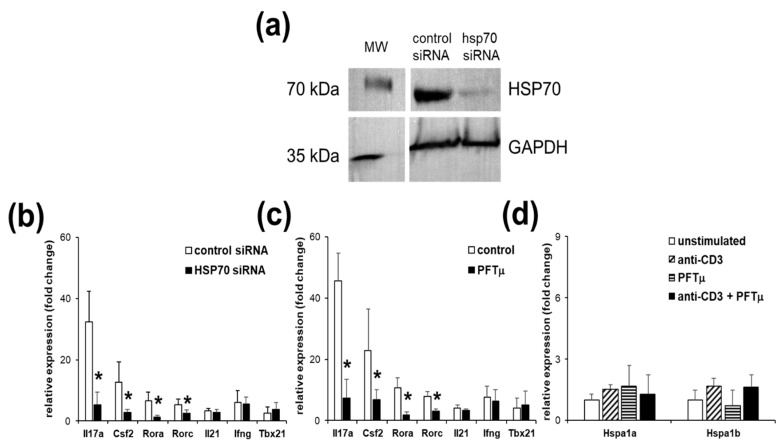
Heat shock protein 70 (HSP70) expression and function manipulation affects Th17 gene expression. (**a**) Western blot analysis of HSP70 and glyceraldehyde 3-phosphate dehydrogenase (GAPDH) proteins in the EL4 TCR^+^ cells that were transfected with HSP70 siRNA or control in relation to the parallel molecular weight (MW) marker separation blot. Representative results from two independent experiments are shown. Gene expression analysis of the EL4 TCR^+^ cells following anti-CD3 stimulation that has been transfected with HSP70 siRNA (**b**) or treated with pifithrin-μ (PFTμ) (10 μM) or with a control (dimethyl sulfoxide; DMSO) (**c**). (**d**) HSP70 gene expression changes of the EL4 TCR^+^ cells following anti-CD3 stimulation or/and PFTμ (10 μM) treatment. Data represent mean +/− SD. * *p* < 0.05, Student’s *t*-test.

We have previously shown that HSP70 presence promotes the development of the Th17 reactions [20]. To further investigate the role of the HSP70 in the regulation of the Th17 responses, we have utilized gene expression in the EL4 TCR^+^ following a manipulation of the HSP70 expression. We have inhibited expression of the inducible forms of HSP70, HSPA1A and HSPA1B with siRNA (Figure 2a). Cell viability has not been different between treated and control transfected cells (not shown). We have found that knockdown of HSP70 resulted in a profound downregulation of all four tested Th17 marker genes, Il17a, Csf2, Rora and Rorc, but neither in the changes of the Il21, nor Ifng and Tbx21 expression, markers of the Th1 response (Figure 2b). 

To further confirm the role of HSP70 in the regulation of the Th17 genes expression in EL4 TCR^+^, we treated these cells with a small molecule inhibitor of HSP70, pifithrin-μ (PFTμ). We have found that presence of the PFTμ strongly inhibited upregulation of the Th17 marker genes but neither Th1 marker genes’ expression nor Il21 (Figure 2c). Cell viability has not been different between treated and control transfected cells (not shown). Hsp70 genes’ expression have not been affected by either anti-CD3 stimulation or/and PFTμ treatment (Figure 2d). Thus, we have demonstrated that functional inhibition of HSP70 results in a suppression of the Th17 genes’ expression following TCR stimulation via binding of CD3, confirming the role of the inducible form of HSP70 in the regulation of the Th17 response.

### 2.3. miRNA Expression Profile of EL4 TCR^+^ Cells

**Figure 3:**
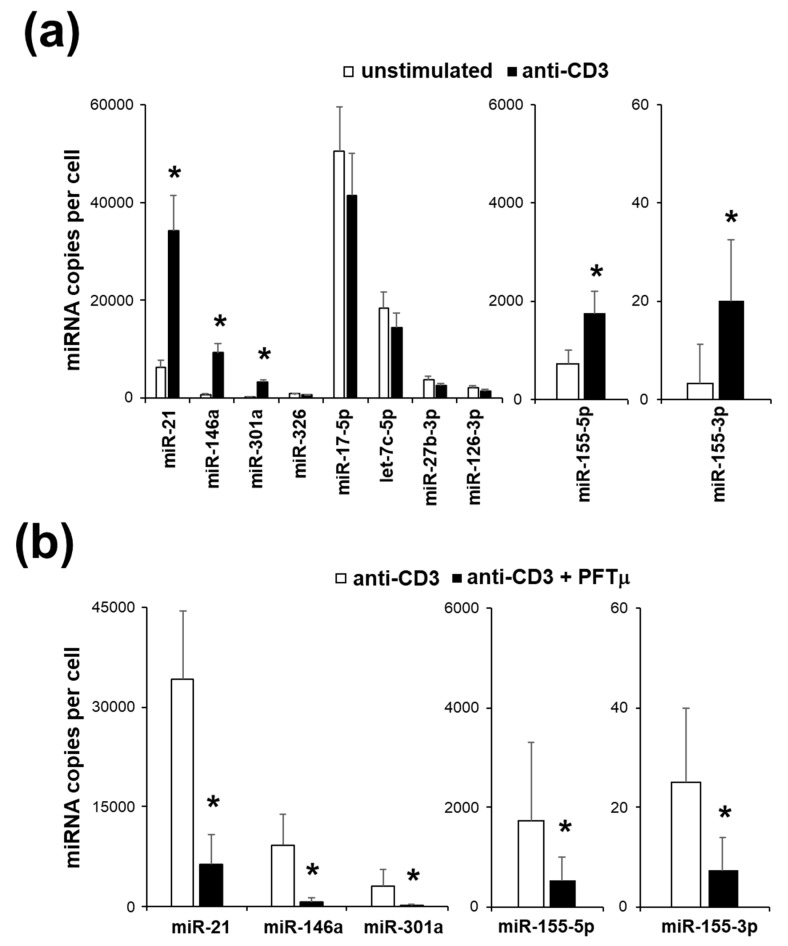
A subset of microRNA (miRNA) is upregulated in EL4 TCR^+^ cells following a T cell receptor (TCR) stimulation. (**a**) Quantification of the miRNA copies per cell in EL4 TCR^+^ cells following an anti-CD3 stimulation. (**b**) Quantification of the miRNA copies per cell in EL4 TCR^+^ cells under anti-CD3 stimulation when treated with PFTμ (10 μM) or with a control (DMSO). Data represent mean +/− SD. * *p* < 0.05, Student’s *t*-test.

To further test the profile of the EL4 TCR^+^, we have assayed changes in miRNA expression following the response to TCR stimulation. We have selected a panel of a miRNA known to be associated with different Th types [26]. Interestingly, we have found that only miRNA that are specific for Th17, miR-21, miR-146a, miR-155-3p, miR-155-5p and miR-301a were upregulated in EL4 TCR^+^ following TCR stimulation (Figure 3a). Thus, miRNA profiling confirms the Th17-like phenotype of the EL4 TCR^+^.

To investigate the role of HSP70 in guiding the changes of the miRNA in EL4 TCR^+^ following TCR stimulation, we have used functional inhibition of HSP70 with a small molecule. PFTμ treatment has led to the significant suppression of the upregulation of the miRNA expression in EL4 TCR^+^ following TCR stimulation (Figure 3b). Cell viability has not been different between treated and control cells (not shown). Thus, we have demonstrated the role of HSP70 in the regulation of the Th17-related miRNA expression following TCR stimulation via binding of CD3.

### 2.4. HSP70 Interacts with a RNA-induced Silencing Complex Protein AGO2

**Figure 4:**
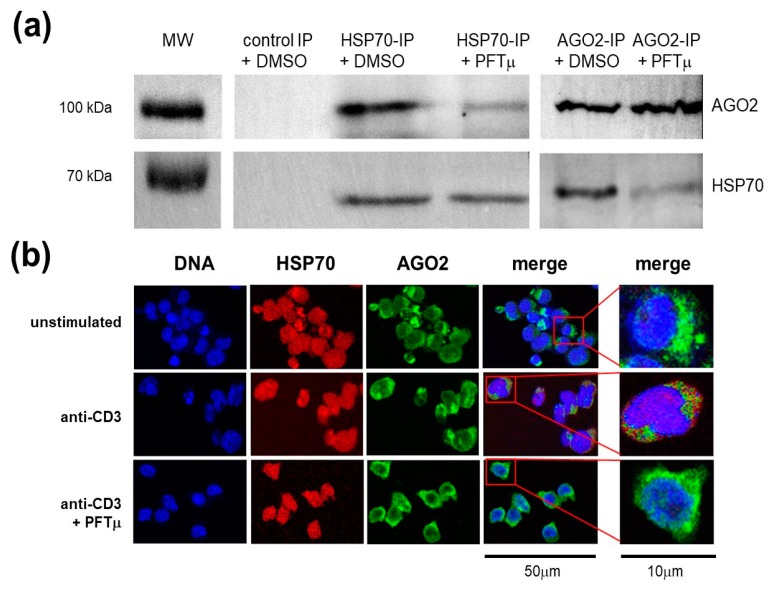
HSP70 is associated with Argonaute 2 protein (AGO2) and RNA-induced silencing complex in EL4 TCR^+^ cells following a TCR stimulation. (**a**) Western blot analysis of AGO2 and HSP70 proteins in the anti-HSP70, in the anti-AGO2 or in the control Ig precipitate of the EL4 TCR^+^ cells that were stimulated with anti-CD3 in the presence of PFTμ (10 μM) or with a control (DMSO) with a parallel separation of the MW marker. Representative results from two to three independent experiments are shown. (**b**) EL4 TCR^+^ cells were cultured for 24 h with or without anti-CD3 stimulation, or with anti-CD3 stimulation in the presence of PFTμ (10 μM) or with a control (DMSO) and assayed for localization of HSP70 and AGO2 proteins by immunofluorescence. Red color represents HSP70 staining, green color represents AGO2 staining whereas blue color demonstrates DAPI staining of the nucleus (scales as depicted). Representative results from two to three independent experiments are shown.

To understand the mechanism of the role of HSP70 in the regulation of the Th17-specific genes’ expression following TCR stimulation, we have performed a series of experiments to localize HSP70 in relation to the RISC, in particular its core component, Argonaute 2 protein (AGO2). First, we have precipitated either HSP70 or AGO2 from the TCR-stimulated EL4 TCR^+^ and found a presence of the AGO2 or HSP70 protein in this precipitate, respectively (Figure 4a). Interestingly, PFTμ treatment has profoundly prevented HSP70 and AGO2 co-precipitaton. Secondly, to confirm the interactions between these two proteins, we have performed an intracellular localization analysis for both HSP70 and AGO2. We have found that HSP70 and AGO2 colocalized after EL4 TCR^+^ activation via TCR (Figure 4b). Furthermore, PFTμ treatment has prevented HSP70 and AGO2 colocalization after anti-CD3 stimulation. Thus, we have demonstrated a close interaction between HSP70 and AGO2 following TCR activation via binding of CD3 in EL4 TCR^+^ cells.

### 2.5. Th17 Specific miRNA Co-Precipitate with HSP70

**Figure 5:**
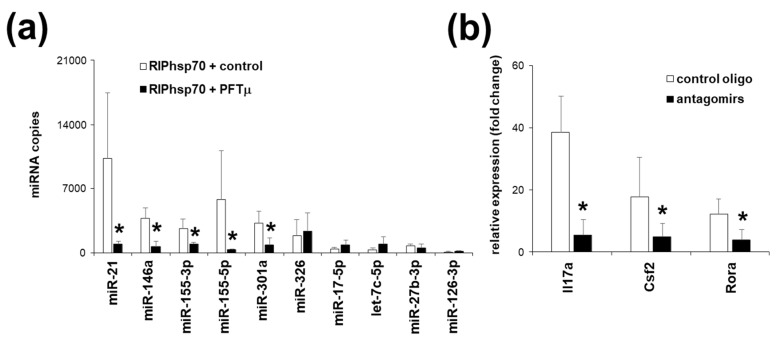
Th17-related subset of miRNA associates with HSP70. (**a**) Quantification of the miRNA copies in the anti-CD3-stimulated EL4 TCR^+^ anti-HSP70 precipitate. (**b**) Gene expression analysis of the EL4 TCR^+^ cells under anti-CD3 stimulation when co-transfected with miR-21, miR-146a, miR-155-3p, miR-155-5p and miR-301a antagomirs or with a control oligonucleotide. Data represent mean +/− SD. * *p* < 0.05, Student’s *t*-test.

To further investigate the role of HSP70 in the RISC complex and in the miRNA function, we have precipitated the HSP70 from TCR-activated EL4 TCR^+^ cells and assayed a presence of small RNA in the precipitate. Indeed, we have found that a significant fraction of miRNA co-precipitated with HSP70. Interestingly, we have found that there is a selective pattern of the miRNA that could be identified to be associated with HSP70 in the TCR-activated EL4 TCR^+^ cells. These miRNA were miR-21, miR-146a, miR-155-3p, miR-155-5p, miR-301a and miR-326, all Th17-related miRNAs (Figure 5a). In contrast, miR-17-5p, let-7c-5p, miR-27b-3p and miR-126-3p have not been found to be present in high copy numbers in HSP70 precipitate, whereas they were present in the AGO2 precipitate (not shown). This suggests a selective pattern of the miRNA that associate with HSP70 in TCR-activated EL4 TCR^+^ cells. Furthermore, when EL4 TCR^+^ cells were activated via TCR in the presence of PFTμ, the majority of the miRNA were significantly less precipitated with HSP70 (Figure 5a). These were miR-21, miR-146a, miR-155-3p, miR-155-5p and miR-301a, but not miR-326.

In order to understand the significance of the expression of the miRNA found to be associated with HSP70, we have transfected the EL4 TCR^+^ cells with miRNA inhibitors, antagomirs, for miR-21, miR-146a, miR-155-5p, miR-155-3p and miR-301a. Our group has already demonstrated that inhibition of each of these miRNA independently inhibited Th17 response [12,13,27]. In the case of the EL4 TCR^+^ cells, TCR stimulation following a combined transfection of antagomirs for miR-21, miR-146a, miR-155-5p, miR-155-3p and miR-301a transfection failed to induce the upregulation of the Th17 marker genes (Figure 5b). Cell viability has not been different between treated and control transfected cells (not shown). Thus, we have demonstrated a significant role of the HSP70-associated miRNA in the regulation of the Th17 phenotype of the EL4 TCR^+^ cells.

### 2.6. HSP70 Inhibition In Vivo Downregulates Experimental Autoimmune Encephalomyelitis

**Figure 6:**
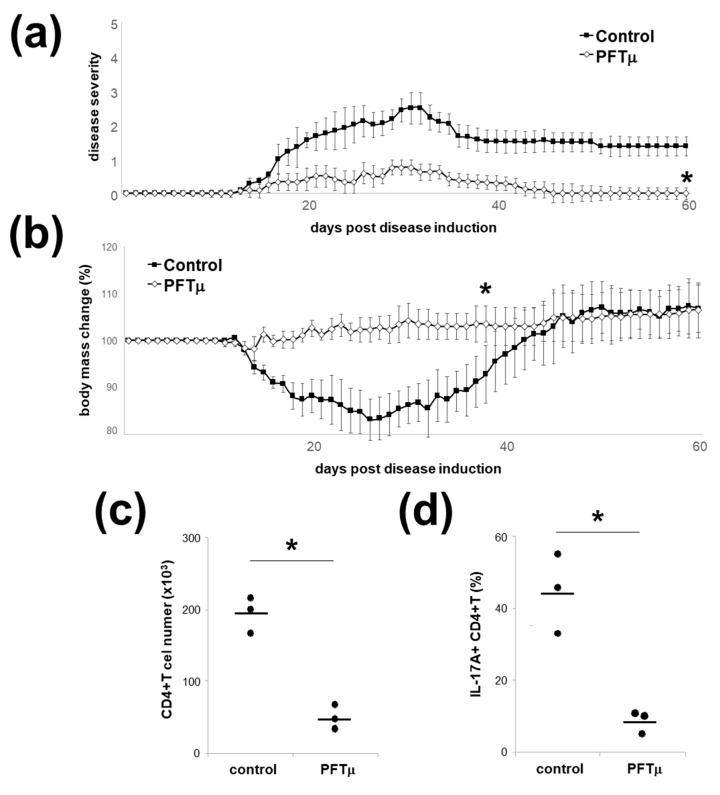
PFTμ in vivo ameliorates experimental autoimmune encephalomyelitis (EAE). (**a**) Clinical scores or (**b**) body mass changes of the mice with EAE that have been treated with PFTμ i.p. on day 0, 2, 4, 6 and 8, or a control (DMSO) are presented (mean ± SEM). Five mice per group were used. * *p* < 0.05, Mann–Whitney *U* test. (**c**) Quantification of CNS-infiltrating CD4+ T cells in PFTμ mice with EAE at 15 days post disease induction. Each dot represents an individual mouse brain; horizontal lines mark the mean. * *p* < 0.05, Student’s *t*-test. (**d**) Quantification of IL-17-producing CNS-infiltrating CD4+ T cells in PFTμ mice with EAE at 15 days post disease induction. Three mice per group have been used, each dot represents an individual mouse CNS; horizontal lines mark the mean. * *p* < 0.05, Student’s *t*-test.

To translate in vivo the findings on the role of HSP70 in directing phenotype of EL4 TCR^+^ cells, we have utilized the MOG35-55-induced EAE in C57Bl/6 mice, a model dependent on the Th17 [2,5]. To this end, we have administered PFTμ to the mice that have been induced for EAE. Interestingly, this treatment has resulted in the amelioration of the clinical signs of the EAE development (Figure 6a) as well as a lack of a significant decrease in body weight during the disease (Figure 6b). In accordance with the clinical data, the mice that received PFTμ had a significant, almost 4-fold decrease in number of CD4+ T-cells in their CNS at 15 days after EAE induction (Figure 6c). Furthermore, the percentage of the IL-17A-producing CD4+ T cells in the CNS of mice that received PFTμ had been significantly reduced (Figure 6d). Thus, we have demonstrated that in vivo HSP70 functional inhibition downregulated the development of the Th17-driven autoimmune process.

## 3. Discussion

In this report, we have demonstrated that HSP70 critically participates in the expression of the Th17 profile genes in the response to the TCR stimulation. Th cell function is tightly controlled by factors encountered in the micro-environment and specifically tailored to successfully orchestrate a response against invading microorganisms and their products. To this end, every Th cell profile has been linked to a specific molecular pathways that guides its generation. With regard to the Th17 differentiation, a specific transcriptional program is induced by IL-6 plus TGF-β, together inducing the transcriptional factors ROR-alpha and ROR-yt [2,3,4]. In addition, IL-21, produced by Th17 cells themselves and, together with TGF-β, also promotes Th17 differentiation [28]. Another essential pathway for Th17 development is IL-1β stimulation [29]. Thus, the IL-6, IL-21, IL-1β and TGF-β signaling have been demonstrated to stimulate Th17 development. However, the mechanisms leading to sustain Th17 cells are not so well known. IL-23 is a cytokine that has been demonstrated to maintain and propagate Th17. This effect results from IL-23 receptor-initiated activation of signal transducer and activator of transcription 3 (STAT3) and inhibition of IL-10 production [30]. Other potential cues aiming at the supporting Th17 profile include Notch [31,32] and JunB [33]. We have already demonstrated that lack of HSP70 expression resulted in downregulation of the Th17 responses as well as resistance to EAE induction [20]. T cell transfer experiments as well as in vitro antigen presentation assays demonstrated that HSP70 deficiency led to an intrinsic dysfunction in the activation of autoreactive T cells with an inhibition of the IL-17 secretion [20]. Here, we provided further evidence for the role of HSP70 in the maintenance of the Th17 phenotype. To this end, we have subcloned a murine cell line, EL4 TCR+, that has been showing a Th17-type of the expression response. With these cells, we have demonstrated that HSP70 manipulation led to a significant and selective modification of the Th17-type expression profile.

HSP70 is a major inducible cytosolic chaperone. It is known to be involved in a number of general activities in the cell, such as binding and release of native and misfolded proteins to favor protein folding cycles [16], transporting unfolded proteins through membranes to enable delivery of cargo to organelles [18], recruiting proteins to the proteasome for turnover [17] and bringing proteins to the endosome/lysosome for chaperone-mediated autophagy [34]. These functions generally control a fate of the damaged proteins. Hence, HSP70 presence and function is particularly enhanced during cell stress, a condition with a high need of a “protein triage” in place. Interestingly, it has been demonstrated that generation of Th17 cells is particularly efficient in the conditions of the cellular stress [35]. This is in stark contrast to other Th effector cell subsets as well as Treg cell differentiation, the polarization of which are either inhibited or not affected under stress conditions [35]. Th17 cells are enriched at sites of chronic inflammation and the local environment has been reported to be able to enhance their differentiation [36]. These environmental cues promoting Th17 are not well understood. With this regard, it is of interest that various stress conditions, including low oxygen, glucose and isotonic stress were all shown to promote Th17 responses [37]. Furthermore, another type of stress, a physiological mechanical damage, through mastication and abrasion, has been demonstrated as a factor responsible for a gingiva Th17 accumulation [38]. Thus, Th17 cells appear as a unique CD4+ T cell subtype induced in a response to various stress conditions. We hypothesize here that the stimulation of the activation of intracellular HSP70 in Th cells in a response to stress conditions is a signal that promotes Th17 response. This mechanism helps Th cells to better sense the environment and tune their responses to the local milieu.

In a search for the mechanisms of the HSP70 control over Th17 gene expression, we have investigated the impact of this protein on the miRNA expression. miRNA emerge as a crucial regulators of the Th17 fate. We and others have previously demonstrated that miR-301a, miR-146a, miR-155-5p, miR-155-3p, miR-21 and miR-326 are factors controlling Th17 development and maintenance, with a particular impact on the generation of the encephalitogenic cells [10,11,12,13,14,15,39]. It is tempting to speculate that these various miRNA are induced through the common mechanisms. Indeed, we have seen that HSP70 presence is required for the proper function of the Th17-promoting miRNAs. We are proposing here that this effect is mediated via interaction of HSP70 with RISC. HSP70 has already been demonstrated as an important component of RISC [40,41,42]. The presence of HSP70 within RISC has been shown to promote proper processing of the miRNA. Adenosine-5′-triphosphate (ATP) hydrolysis by HSP70 is required for RISC assembly of small RNA duplexes [43]. Thus, HSP70 is known to be present within the RISC and facilitates the processing and function of miRNA. Indeed, we have demonstrated that HSP70 co-localized intracellularly with the AGO2, a critical protein for the miRNA function and RISC, upon TCR stimulation, by two independent methods, co-immunoprecipitation and intracellular imaging. This supports recently published data that HSP70, via direct interaction, render AGO2 into an open, active form [44]. Furthermore, HSP70 presence and function appeared to be critical for a subset of the miRNAs. Intriguingly, these miRNAs were largely those that have been linked to the Th17 profile promotion. Thus, we are proposing a model of two modes of miRNA action in Th cells, the first is HSP70-dependent and promotes Th17-associated miRNA and the second one, HSP70-independent, that leads to generation of the other miRNA. This way, HSP70 presence within the RISC would not only help to generate a functional protein-miRNA complex, but also to favor the Th17, promoting miRNA presence within this complex. We believe such mechanism would fit into the concept of the stress response, with HSP70 playing a switch between stress-related and housekeeping-related miRNA programs.

Th cells can simultaneously express transcriptional factors and cytokines characteristic of distinct polarized subsets, a phenomenon described as plasticity [45]. Th17 subtype has been particularly know to have a high plasticity potential. It has been demonstrated that expression of the factors like c-maf, B lymphocyte-induced maturation protein-1, eomesodermin or aryl hydrocarbon receptor can lead to skewing of the Th17 cells toward less or more pathogenic cell types [46,47,48,49]. Furthermore, Rbpj and JunB expression have been demonstrated to stimulate the expression of the IL-23 receptor gene, rendering the Th17 cell fully mature and allowing their expansion [32,33]. Our study indicates that HSP70 might be another molecule involved in the Th17 lineage support. One mechanism of the HSP70 action on Th17 is via the support of the miRNA generation that promotes this Th cell pattern. Interestingly, another pathway can also contribute to this effect. Our group have previously reported on the role of HSP70 in regulation of the Notch1 pathway in Th cells [50]. We found that HSP70 was required for effective Notch signaling in activated Th cells, providing a help in the effective release and function of the intracellular domain of Notch (NICD). NICD activates transcription of Notch target genes with the transcription factor Rbpj, a factor known to stabilize the Th17 subset. Thus, HSP70-miRNA and HSP70-Notch-Rbpj pathways may represent mechanisms of HSP70 control over the Th17 gene expression program.

Our data have demonstrated that HSP70 downregulation led to a marked suppression of the Th17 marker genes’ expression. Since HSP70 intracellular levels are a good indicator of the stress condition, the HSP70 inhibition could help to relieve the pro-immune reactivity, as if the stress has been resolved. Our in vivo data on amelioration of EAE with HSP70 functional inhibition support the concept that this protein manipulation would tackle pathogenic Th17. However, in vivo inhibition of EAE by blocking HSP70 function may have also resulted from non-Th17 cell effects. To this end, we and others have already shown that antigen-presenting cells’ (APC) manipulation of HSP70 leads to significant changes of the major histocompatibility complex class II presentation and subsequent modulation of Th cell activation [51,52,53]. Furthermore, our group have shown a role of HSP70-peptide complexes in the activation of NK cells that affected the course of autoimmunity [54]. It has also been recently reported that HSP70 overexpression led to Fas downregulation and decreased IL17A secretion by Th cells, whereas systemic in vivo knockdown of the HSP70 in EAE animals counterbalanced miR-374c-induced upregulation of IL17A in splenic Th cells [55]. Although that paper only analyzed IL17A secretion, and not the extended spectrum of the Th17-type response, it implicated another mechanism of the HSP70 impact on the Th cell function, via Fas modulation. Further studies are warranted to unravel the full spectrum of the systemic HSP70 function inhibition and whether they could be translated into the treatment of autoimmune demyelination.

**Figure 7:**
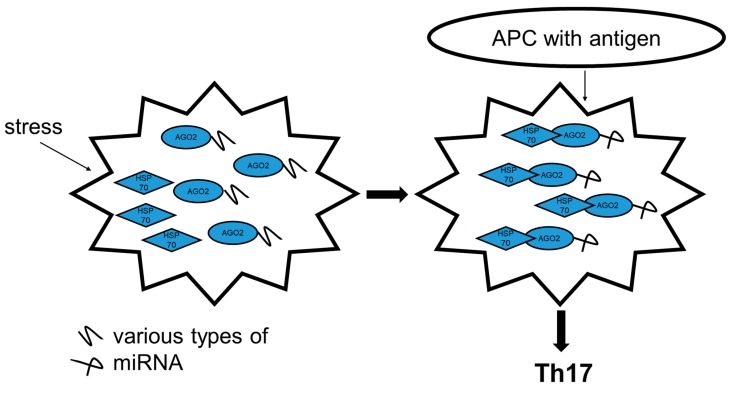
Proposed model of HSP70’s impact on Th cell fate. In conditions of stress, HSP70 is activated and forms a complex with AGO2. This complex has a different propensity for miRNA processing, which under the TCR stimulation by a cognate antigen presented by APC, promotes generation of pro-Th17 miRNA. As a result, the Th cell turns into pathogenic Th17 with a capacity to initiate autoaggression.

In conclusion, our results support a model in which high levels of HSP70 directly promote Th17 genes’ expression after TCR stimulation. This effect results from a direct intracellular interaction of HSP70 with a RISC complex (Figure 7). The activity of HSP70 in the Th17 promotion is dependent on the regulation of the expression of a specific set of miRNA. Selective inhibition of these miRNA or direct blockage of HSP70 function downregulates Th17 genes’ expression. To translate these findings in vivo, we managed to downregulate EAE development by in vivo administration of the HSP70 small molecule inhibitor. These data suggest a potential for HSP70-targeted therapies in MS. Unfortunately, the clinical application of PFTμ is hindered owing to the poor solubility, low stability and selectivity. Fortunately, it has recently been shown that nanotechnology-based delivery systems can significantly improve the pharmacokinetics and biodistribution of PFTμ [56]. Hopefully, these approaches can be developed further, allowing for more efficient and more precise targeting of the HSP70 function and better tackling of the autoimmune demyelination.

## 4. Materials and Methods 

### 4.1. Cell Culture, Cell Sorting and Reagents

EL4 cell line cells (Sigma-Aldrich, St. Louis, MO, USA) were cultured in Dulbecco’s Modified Eagle Medium (DMEM) supplemented with 10% fetal bovine serum (FBS), 2 mM glutamine, 100 U/mL penicillin and 100 mg/mL streptomycin (all reagents from Sigma-Aldrich, St. Louis, MO, USA). EL4-TCR^+^ have been subcloned from TCR-positive EL4 cells and cultured in DMEM supplemented with 10% FBS, 2 mM glutamine, 100 U/ml penicillin and 100 mg/mL streptomycin. Cell sorting was performed following a flow cytometry analysis with BD Aria equipment (Beckton Dickinson, BD Biosciences, San Jose, CA, USA) according to standard procedures, using an anti-mouse TCRβ chain antibody (H57-597; BD Biosciences, San Jose, CA, USA). EL4-TCR^+^ were subsequently kept in culture for at least 7 days without losing CD3 expression. Cells were stimulated with plate-bound anti-CD3 (5 μg/mL) (145-2C11; BD Biosciences, San Jose, CA, USA) with parallel unstimulated cultures as controls. PFTμ compound was procured from Sigma-Aldrich (Sigma-Aldrich, St. Louis, MO, USA). The compound was dissolved in dimethyl sulfoxide (DMSO) (Sigma-Aldrich, St. Louis, MO, USA) for in vitro and in vivo experiments and used at indicated concentrations. DMSO alone was used as a control stimulation. Cell viability was assayed with flow cytometry using a propidium iodide (PI) staining (Sigma-Aldrich, St. Louis, MO, USA).

### 4.2. Mice

All mice used were adults, aged 6 to 12 weeks. C57BL/6J mice were derived from the Jackson Laboratory. Mice were maintained in the animal facilities of Medical University of Lodz. All experiments involving animals were done in compliance with the relevant laws and institutional guidelines and were approved by the ethics committees.

### 4.3. Western Blot Analysis

Cells were lysed and total lysates were resolved on sodium dodecyl sulfate electrophoresis gels by standard procedures. Immunoblotting was performed with mouse primary antibodies to HSP70 or AGO2 (sc-24 and B-3 respectively, both from Santa Cruz Biotechnology, Dallas, TX, USA) and glyceraldehyde-3-Phosphate Dehydrogenase (GAPDH) (MAB374; Merck Millipore, Burlington, MA, USA), and visualized by G: BOX Chemi (Syngene, Cambridge, UK).

### 4.4. Co-immunoprecipitation and RNA-binding Protein Immunoprecipitation (RIP)

To assess protein interactions between HSP70 and AGO2, an immunoprecipitation (IP) assay was performed. For IP, 5 μg of anti-hsp70 antibody (sc-24; Santa Cruz Biotechnology, Dallas, TX, USA) was used. To determine the total miRNAs associated with HSP70 protein, a RIP assay (Magna RIP; Merck Millipore, Burlington, MA, USA) was performed according to the manufacturer’s protocol. For RIP, 5 μg of anti-HSP70 antibody (sc-24; Santa Cruz Biotechnology, Dallas, TX, USA) or 5 μg of anti-AGO2 (clone 2A8, Merck Millipore, Burlington, MA, USA) antibody and 5 μg normal mouse IgG2a (negative control, Santa Cruz Biotechnology, Dallas, TX, USA) were conjugated to Magnetic Beads Protein A/G. After, IP protein digestion by proteinase K was performed and RNA was purified according to the manufacturer’s protocol. The profile of total RNA associated with HSP70 was determined by a 2100 Bioanalyzer System (Agilent Technologies, Santa Clara, CA, USA) using Pico RNA Assays (Agilent Technologies, Santa Clara, CA, USA).

### 4.5. Th17 In Vitro Differentiation

Naive CD4^+^ T cells were obtained from a C57Bl/6 mice spleen with a magnetic cell sorting using a Naive CD4^+^ T Cell Isolation Kit (Miltenyi Biotec, Bergisch Gladbach, Germany). Th17 in vitro differentiation was performed from naive CD4^+^ T cells that had been stimulated with plate-bound anti-CD3 (5 μg/mL) and anti-CD28 (10 μg/mL) (37.51; BD Biosciences, San Jose, CA, USA), polarized for 6 days with the following: TGFβ (2 ng/mL), IL-1β (20 ng/mL), IL-23 (20 ng/mL) in the presence of anti-IL-4 (10 μg/mL) (11B11; BD Biosciences, San Jose, CA, USA) and anti-IFNγ (10 μg/mL) (XMG1.2; BD Biosciences, San Jose, CA, USA) and with the addition of 40 mM NaCl for Th17. TGFβ (10 ng/mL) and IL-2 (100 U/mL) in the presence of anti-IL-4 (10 μg/mL) and anti-IFNγ (10 μg/mL). Following 6 days of culture in Th17, cells were assayed for RNA expression.

### 4.6. Transfections of Antagomirs against miR-21, miR-146a, miR-155-5p, miR-155-3p and miR-301a and HSP70 Expression Silencing

Antagomirs against miR-21, miR-146a, miR-155-5p, miR-155-3p and miR-301a and control antagomir (scrambled oligonucleotide) were purchased from Exiqon Inc. (Exiqon Inc., Vedbæk, Denmark). siRNA for HSP70 (for Hspa1a, assays IDs: 221289, 221290, 221291; for Hspa1b, assays IDs: s201487, s201486, s201488) and control oligonucleotides (Silencer Select Negative Control #1 and #2 siRNA) were purchased from ThermoFisher Scientific (Thermo Scientific, Waltham, MA, USA). EL4 TCR^+^ cells were transfected by electroporation with 4D-Nucleofector (Lonza, Basel, Switzerland) according to the manufacturer’s instructions.

### 4.7. Extraction of RNA and mRNA Expression Analysis

EL4-TCR^+^ were stimulated with plate-bound anti-CD3 (5 μg/mL) for 72 h with parallel unstimulated cultures as controls. Extraction of total RNA was performed using a mirVana miRNA isolation Kit (ThermoFisher Scientific, Waltham, MA, USA). For quantitative analysis of RNA expression, we carried out real-time quantitative polymerase chain reaction (qPCR) with TaqMan probes (Life Technologies, ThermoFisher Scientific, Waltham, MA, USA), using a 7500 Real-Time PCR System (Applied Biosystems, Foster City, CA, USA). mRNA expression data were normalized to that of the DNA-directed RNA polymerase I subunit RPA1 (POLR1A). Expression was evaluated by the comparative cycling threshold (ΔΔCT) method.

### 4.8. miRNA Quantification

Absolute quantification of miRNA in RNA samples was performed using a reverse-transcriptase droplet digital PCR (ddPCR) method with ddPCR Supermix for Probes (Bio-Rad, Hercules, CA, USA) using a specific miRNA TaqMan gene expression probe (Life Technologies, ThermoFisher Scientific, Waltham, MA, USA) and a QX200 Droplet Generator, a QX200 Droplet Reader, and a PX1 PCR Plate Sealer all from Bio-Rad (Bio-Rad Hercules, CA, USA), according to the manufacturer’s instructions. The results have been expressed as miRNA copies number per cell or per sample volume.

### 4.9. Immunocytochemistry

For immunofluorescence analysis, cells were transferred from U-bottom plates to gelatin-coated microscope slides by cytospine (300xg, 10 min) and perfused by PBS containing 4% (*w/v*) paraformaldehyde for 20 min at room temperature. Fixed cells were washed with PBS and blocked with 10% rabbit normal blocking serum (Santa Cruz Biotechnology, Dallas, TX, USA), supplemented with 0.3% Triton X-100 (Sigma-Aldrich, St. Louis, MO, USA) for 45 minutes at RT. Next, cells were washed and double-stained for HSP70 (rabbit polyclonal antibody, 1:500, SPA-812, Enzo Life Sciences, Inc., Farmingdale, NY, USA) and AGO2 (mouse monoclonal antibody, 1:500, B-3, Santa Cruz Biotechnology, Dallas, TX, USA) with PBS supplemented with 1.5% of normal blocking rabbit serum and 0.3% Triton X-100, overnight at 4 °C in wet chamber. Subsequently, cells were washed and secondary fluorescent antibody (goat anti-rabbit IgG-TR and goat anti-mouse IgG-FITC; 1:100 both Abcam, Cambridge, UK), supplemented with 1.5% of normal blocking rabbit serum and 0.3% Triton X-100 was added for 1 h at RT. For fluorescent DNA nuclei staining, DAPI was used (1.5 mg/mL UltraCruz Mounteining Medium, Santa Cruz Biotechnology, Dallas, TX, USA). The intracellular colocalization of AGO2 and HSP70 was analyzed by confocal microscopy (Nikon D-Eclipse C1) using EZ-C1 software.

### 4.10. Induction of EAE and PFTμ Treatment

We induced active EAE by subcutaneous immunization over the abdominal flanks of 8- to 12-week-old mice with 0.15 mg MOG35-55 peptide in 150 uL CFA containing 0.75 mg M. tuberculosis. In addition, 0.2 ug Pertussis toxin (Sigma) was injected i.v. on days 0 and 2. PFTμ has been administrated i.p. in the dose of 200 μg per injection (200 μL) per mouse on days 0, 2, 4, 6 and 8 post EAE induction. As a control, 200 μL of solvent (DMSO) was injected i.p. in the same fashion. Mice were checked daily for body weight and signs of EAE and scored on a scale of 0–5 as follows: 0, no disease; 1, weak tail or unsteady gait; 2, hind-limb paresis; 3, hind-limb paralysis; 4, hind- and fore-limb paralysis; 5, death or euthanasia for humane reasons. Weight of mice was also checked daily and changes were assessed in relation to individual mouse weight at day 1 (percent of weight change). 

### 4.11. Isolation of CNS-infiltrating Cells and Analysis of Infiltrating CD4+ T Cells

Mice were perfused intracardially with PBS before dissection of the brain and spinal cord, which were subsequently homogenized and brain-infiltrating mononuclear cells were isolated using 37%/70% Percoll gradients. For intracellular detection of cytokine production, CNS-infiltrating mononuclear cells were stimulated with 500 ng/mL phorbol dibutyrate and 500 ng/mL ionomycin in the presence of brefeldin A for 6 h. Detection of IL-17A+ CD4+ T cells was performed by intracellular staining using a Mouse Regulatory T cell Staining Kit (ThermoFisher Scientific, Waltham, MA, USA), using an anti-mouse IL17A antibody (TC11-18H10; BD Biosciences, San Jose, CA, USA). Four-color flow cytometry analysis was performed with the LSR II (BD Biosciences, San Jose, CA, USA) according to standard procedures. Flow cytometry data were analyzed with FlowJo.

### 4.12. Statistical Analysis

Results were compared using the program, StatGraphics Centurion XV (StatPoint Technologies, Warrenton, VA, USA). Clinical data from mice with EAE were compared using the Mann–Whitney *U* test. Results of qPCR and ddPCR were compared using the Student’s *t*-test. *p*-values < 0.05 were considered statistically significant.
